# Detection of severe aortic stenosis by clinicians versus artificial intelligence: A retrospective clinical cohort study

**DOI:** 10.1016/j.ahjo.2024.100485

**Published:** 2024-11-22

**Authors:** Geoffrey A. Strange, Michael P. Feneley, David Prior, David Muller, Prasanna Venkataraman, Yiling Situ, Simon Stewart, David Playford

**Affiliations:** a Institute for Health Research, University of Notre Dame Australia, Fremantle, WA, Australia; b Faculty of Medicine and Health, The University of Sydney, Sydney, NSW, Australia; c St Vincent's Hospital, Sydney, NSW, Australia; d St Vincent's Hospital, Melbourne, VIC, Australia; e School of Medicine, Dentistry & Nursing, University of Glasgow, Glasgow, United Kingdom; f Faculty of Medicine, University of Melbourne, Melbourne, VIC, Australia

**Keywords:** Aortic stenosis, Artificial intelligence, Echocardiography, Clinical management

## Abstract

Many severe aortic stenosis (AS) cases are undetected and/or not considered for potentially life-saving treatment, with a persistent male-bias reported among those undergoing aortic valve replacement (AVR). We evaluated the clinical value of a validated artificial intelligence automated alert system (AI-AAS) that detects severe AS from routine echocardiographic measurements. In a retrospective, clinical cohort of 21,749 adults investigated with transthoracic echocardiography at two tertiary-referral centres, we identified 4057 women (aged 61.6 ± 18.1 years) and 5132 men (60.8 ± 17.5 years) with native aortic valves. We firstly applied the AI-AAS to the cardiologists' reported echo measurements, to detect all AS cases, including guideline-defined severe AS. Two expert clinicians then independently reviewed the original clinical diagnosis/management based on the initial report. Initially, 218/9189 (2.4 %, 95%CI 2.1–2.7 %) severe AS cases were diagnosed. The AI-AAS subsequently increased this number by 158 (52 % women) to 376 cases (4.1 %, 95%CI 3.7–4.5 %) of severe guideline-defined AS. Overall, more women were under-diagnosed (92/169 [54.4 %] versus 80/207 [38.6 %] men – adjusted odds ratio [aOR] 0.21, 95%CI 0.10–0.45). Even when accounting for potential contraindications to valvular intervention, women were persistently less likely to be considered for valvular intervention (aOR 0.54, 95%CI 0.31–0.95) and/or underwent AVR (aOR 0.29, 95%CI 0.09–0.74). Our study suggests an AI-AAS application that is agnostic to gender, haemodynamic bias, symptoms, or clinical factors, provides an objective alert to severe forms of AS (including guideline-defined severe AS) following a routine echocardiogram, and has the potential to increase the number of people (especially women) directed towards more definitive treatment/specialist care.

## 1 Introduction

Aortic stenosis (AS) is the most common form of valvular heart disease managed in clinical practice [1]. It is estimated that at least 1.5 % of people aged 55 years have severe AS [2]. Incidence of severe AS rises steeply with age and among those with high levels of antecedent risk for heart disease [3]. If untreated, there are substantial societal costs attributable to high rates of premature mortality and quality-adjusted-life-years lost across the spectrum of moderate to severe disease [2]. Beyond ensuring health systems can deliver potentially life-saving surgical [4,5] and transcatheter [6] aortic valve replacement/intervention (SAVR/TAVi) procedures [7], those presenting with severe forms of AS need to be rapidly identified and then optimally managed according to expert recommendations [4]. However, even within the most well-resourced healthcare settings, this is not happening [5]. Moreover, despite evidence that men and women are equally at risk of developing severe AS [3], there persists a predominance of males within large observational studies [8] and trial cohorts [9] focusing on those being treated for severe AS. Thus, calls for automated systems to alert clinicians that severe AS is present when echocardiography is performed and reported have been made [10]. Various systems that synthesise clinical data from a broad range of sources (from cardiac auscultation to in situ mechanical sensors) have been evaluated in the setting of AS with varying success [[10], [11], [12], [13], [14], [15]].

Herewith, we evaluated the clinical potential of a previously validated artificial intelligence automated alert system (AI-AAS [16]) to augment the identification of severe AS. We hypothesised that compared to routine clinical management, the AI-AAS would identify more cases of severe AS (including those with guideline-defined severe AS [17,18]). We then modelled the potential of AI-AAS to direct both men and women to more definitive evaluation for treatment if the automated alert triggered a simple clinical pathway/referral to the heart care team.

## 2 Materials and methods

### 2.1 Study design

This was a retrospective, multicentre, clinical cohort study focussing on every patient undergoing routine echocardiography at two tertiary referral hospitals in Australia. The AI-AAS independently assessed all echo reports (excluding text and conclusions) to identify all AS cases (without any clinical input), followed by cardiologist's evaluation of the initial AS diagnosis and clinical management based on the same echo reports and medical records. Where appropriate, this study conforms to the “Standards for Reporting Diagnostic accuracy studies” (STARD) and RECORD guidelines for the reporting of routine clinical data [19]. Ethics approval was obtained under the auspices of the National Echo Database of Australia (NEDA – ACTRN12617001387314).

### 2.2 Study cohort

**Fig. 1:**
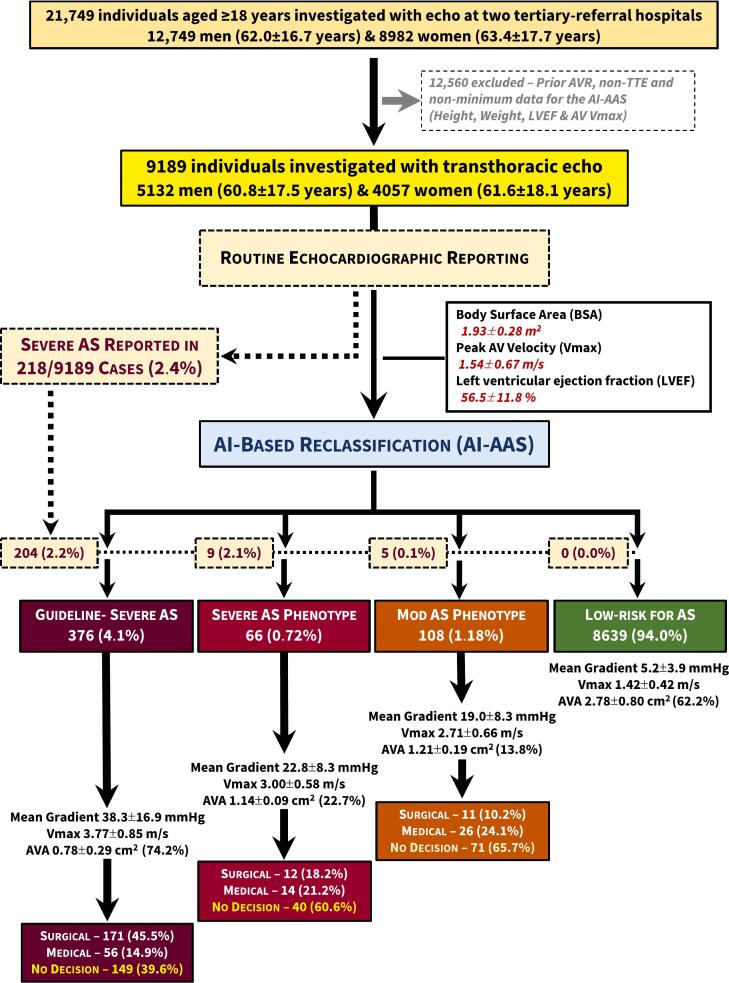
Flow chart according to the initial human classification and subsequent AI-AAS reclassification of cases. Legend: Aortic stenosis, AS; aortic valve area, AVA.

Individuals aged ≥18 years presenting to the echocardiographic laboratories of St Vincents' Hospitals in Melbourne and Sydney, Australia during 2018–2021 were identified. Patient consent was obtained by a combination of authorised consent waiver for retrospective data collection and prospective opt-out consent. We excluded cases undergoing transesophageal echo, exercise and pharmacological stress echo, myocardial contrast echo, point-of-care ultrasound (POCUS), repeat echo studies, and with a prior aortic valve (AV) intervention. We further identified those individuals with sufficient information (height, weight, peak AV velocity [Vmax] and left ventricular ejection fraction [LVEF]) for the AI-AAS to accurately identify [16] mild-to-severe AS without the need for a documented aortic valve area (AVA) Fig. 1.

### 2.3 Data sources

The primary data source was the echo database located at each site plus the medical records of those identified with any form of AS. A backup copy of the echocardiographic report database at each site was taken. The individual echocardiographic measurement and report data were extracted using a standardised extraction tool we have developed and previously published (Strange et al. AHJ 2018). Echocardiographic data from both sites were combined with a unique identifying code for each site.

### 2.4 Artificial intelligence classification of aortic stenosis

The AI-AAS independently scrutinised the same basic demographic (age and sex), anthropometric (height and weight) and cardiologist confirmed echocardiographic measurement data to derive a probability score (between 0 and 1) for the likelihood of severe AS. The AI-AAS training and validation in two separate has been reported previously [16,17]. Briefly, the AI-AAS was trained via a modified Mixture Density Network model in 442,276 individuals (70 %) and their 754,503 echocardiograms randomly selected from a large clinical cohort [10,16]. The capacity of the AI-AAS to detect severe AS was then validated in the residual cohort comprising 189,548 individuals and their 322,642 echocardiograms. The AI outputs, validated with mortality outcomes, consist of four groups. Group 1 and 2: High Risk of the severe AS phenotype (1) meeting current clinical guidelines and (2) those with high risk, but not meeting current guidelines for severe AS; Group 3: Moderate Risk of Severe AS, Group 4: Low Risk of Severe AS [17].

### 2.5 Initial clinical diagnosis & management

Cardiologists with sub-speciality expertise in echocardiography were given a list of AI-AAS detected AS cases, but were blinded to severity classification. They scrutinised the echo image and measurement outputs, the echocardiographic reports and hospital medical records, and completed standardised case-report forms. This included the cardiologist-reported AS severity along with clinical data, including concurrent clinical diagnosis, symptom and pharmacotherapy immediately following the index echo (last recorded for that individual). Any hospitalisations occurring up to a census date of December 31, 2022 were also documented. The primary diagnosis, type of investigation (e.g., coronary angiography as part of a surgical work-up protocol) and procedure (including SAVR, TAVi, balloon aortic valvuloplasty [BAV], other valve intervention, percutaneous intervention and/or coronary artery by-pass surgery) and length of stay were recorded for each episode. Subsequently, in consultation with an independent cardiologist (DPR), they then documented the following for everyone – 1) Recognition/diagnosis of severe AS (noting concordance/discordance with the AI-AAS output), and 2) Intended clinical management (i.e., AV intervention or medical [conservative] management). Finally, those without evidence of definitive clinical management were identified as the “non-definitive management” group.

### 2.6 Study outcomes

The primary outcome was the proportion of cases identified with guideline-defined severe AS [15] as identified by the AI-AAS compared with the initial cardiologist echo report. Secondary outcomes were the proportion of cases with moderate or severe AS outside of clinical practice guidelines identified by AI-AAS, the documented management plan and subsequently implemented (pattern of hospital activity) management for anyone in each AI-AAS grouping. As above, three main categories were generated – valve intervention (i.e., planned SAVR, TAVi and/or BAV), medical management (i.e., no valve intervention, pharmacological therapy) or no definitive plan.

### 2.7 Statistical analyses

With ~20,000 eligible individuals with echocardiograms, based on our previous studies [16,20] we expected that half would have a documented Vmax (minimum requirement for the AI-AAS) and a minimum of 2.5 % with guideline-defined severe AS. Thus, we expected to identify >250 (95 % confidence intervals [CI] 220–283) cases, with an additional 1.5 % with moderate AS. Standard methods for descriptive statistics, including means (± SD), median (interquartile range, IQR) and proportions (with 95%CI) were performed. Between-group comparisons included ANOVA, Student's *t*-test and Chi-square analyses where appropriate. Multiple logistic regression (entry model) was used to determine the correlates of initial recognition of severe AS –according to age, sex, body mass index, Vmax and LVEF and then the full parameters presented in Table 1 to generate adjusted odds ratios (OR) and 95%CI. The same method (based on the parameters presented in Table 2, including initial AS status) was used to identify the correlates of the three specified categories of management. All analyses were performed with SPSS v28.0 (IBM Corporation Chicago, IL) and statistical significance accepted at a two-sided alpha of 0.05.

**Table 1:** Cohort characteristics according to initial human echo report (N = 550).

	Severe AS (human) N = 218		Non-severe AS (human) N = 332	
	Men (n = 131)	Women (n = 87)	Men (n = 146)	Women (n = 186)
Demographic profile				
Age, years	75.3 ± 10.5	80.3 ± 9.4	75.6 ± 12.1	76.7 ± 11.1

Clinical profile				
BMI, kg/m^2^	28.5 ± 5.0	27.9 ± 5.7	27.7 ± 5.2	29.4 ± 7.5
Systolic BP, mmHg	129 ± 21	140 ± 26	132 ± 26	139 ± 28
Heart rate, bpm	73 ± 19	73 ± 14	71 ± 15	71 ± 13

Aortic valve function/dimension				
Severe AS (guidelines)	127 (96.9 %)	77 (88.5 %)	80 (54.8 %)	92 (49.5 %)
Vmax, m/s	4.16 ± 0.62	4.17 ± 0.68	3.05 ± 0.74	2.97 ± 0.74
Mean AV gradient, mmHg	45.5 ± 13.8	46.8 ± 17.1	25.3 ± 11.9	23.1 ± 11.5
Valve area, cm^2^	0.83 ± 0.26	0.77 ± 0.25	1.08 ± 0.33	1.02 ± 0.24

Right ventricular function				
eRVSP, mmHg	37.1 ± 13.8	38.6 ± 11.2	37.6 ± 13.9	39.7 ± 14.1
TR peak velocity, m/s	2.79 ± 0.47	2.85 ± 0.51	2.81 ± 0.55	2.89 ± 0.55

Left ventricular function/dimensions				
LAVi, mL/m^2^	47.6 ± 18.5	51.8 ± 22.1	49.2 ± 22.3	49.8 ± 20.6
LVDD, cm	4.92 ± 0.74	4.50 ± 0.69	4.83 ± 0.74	4.24 ± 0.68
LVSD, cm	3.55 ± 0.95	3.11 ± 0.83	3.67 ± 0.85	2.97 ± 0.74
LVMi, g/m^2^	119 ± 28.1	112 ± 33.4	111 ± 30.3	95.9 ± 29.4
LVEF, %	54 ± 15	57 ± 16	50 ± 15	59 ± 12
Septal E:e′ ratio	18.1 ± 8.21	21.3 ± 9.63	18.7 ± 10.6	20.4 ± 11.2
Transmitral E:A ratio	1.01 ± 0.69	1.12 ± 0.75	1.05 ± 0.79	1.07 ± 0.75
SVi, mL/m^2^	38.2 ± 12.7	40.8 ± 13.4	37.3 ± 14.0	40.2 ± 13.3

Legend: This table compares the characteristics of those 218 men and women identified with guideline-defined severe aortic stenosis (AS) versus the rest (n = 332) identified by the AI-AAS as having moderate or greater AS. The latter includes 14 cases initially reported by the human to have severe AS but did not meet guideline criteria. Incomplete data for – systolic blood pressure, BP (253 cases); heart rate (454 cases); mean aortic valve (AV) gradient (509 cases); AV area, AVA (340 cases); estimated right ventricular systolic pressure (eRVSP)/tricuspid regurgitation (TR) peak velocity (408 cases); left atrial volume index, LAVi (404 cases); left ventricular diastolic dimension, LVDD/left ventricular systolic dimension, LVSD (394/387 cases); left ventricular mass index, LVMi (284 cases); E:e′ ratio (399 cases); E:A ratio (415 cases) and stroke volume index, SVi (449 cases); left ventricular ejection fraction, LVEF.

**Table 2:** Cohort characteristics according to AI-derived reclassification (N = 9189).

	High risk AS phenotype Guideline-severe AS		High risk AS phenotype Guideline non-severe AS		Moderate risk AS phenotype		Low-AS risk group	
	Men (n = 207)	Women (n = 169)	Men (n = 19)	Women (n = 47)	Men (n = 51)	Women (n = 57)	Men (n = 4855)	Women (n = 3784)
Demographic profile								
Age, years	75.7 ± 11.0	78.9 ± 10.9	73.0 ± 14.7	76.9 ± 10.8	75.6 ± 11.5	75.6 ± 9.9	60.4 ± 17.9	60.0 ± 17.9

Clinical profile								
BMI, kg/m^2^	28.4 ± 4.8	28.8 ± 6.3	25.6 ± 5.0	29.2 ± 9.0	27.6 ± 6.0	29.1 ± 7.1	28.3 ± 6.1	28.1 ± 7.4
Systolic BP, mmHg	132 ± 24	139 ± 23	120 ± 31	145 ± 27	129 ± 21	135 ± 36	131 ± 22	131 ± 24
Heart rate, bpm	73 ± 18	71 ± 13	67 ± 13	77 ± 15	71 ± 14	70 ± 12	71 ± 15	74 ± 14

Aortic valve function/dimension								
Vmax, m/s	3.82 ± 0.79	3.69 ± 0.90	3.04 ± 0.69	2.99 ± 0.54	2.77 ± 0.68	2.65 ± 0.65	1.41 ± 0.45	1.44 ± 0.34
Mean AV gradient, mmHg	39.4 ± 15.6	37.0 ± 18.4	23.3 ± 10.8	22.6 ± 7.40	20.4 ± 8.74	17.7 ± 7.78	5.19 ± 4.31	5.12 ± 3.29
Valve area, cm^2^	0.89 ± 0.31	0.85 ± 0.22	1.10 ± 0.06	1.14 ± 0.09	1.16 ± 0.12	1.18 ± 0.09	3.11 ± 0.89	2.46 ± 0.63

Right ventricular function								
eRVSP, mmHg	36.6 ± 10.9	38.1 ± 11.7	44.0 ± 26.2	41.0 ± 12.6	37.3 ± 11.1	41.5 ± 16.9	30.9 ± 10.3	30.4 ± 10.5
TR peak velocity, m/s	2.77 ± 0.46	2.83 ± 0.50	3.00 ± 0.89	2.95 ± 0.50	2.79 ± 0.49	2.95 ± 0.64	2.50 ± 0.47	2.48 ± 0.48

Left ventricular function/dimensions								
LAVi, mL/m^2^	48.7 ± 20.0	48.1 ± 19.6	50.3 ± 14.2	53.4 ± 22.8	47.6 ± 25.8	55.6 ± 23.4	36.1 ± 15.0	34.0 ± 15.6
LVDD, cm	4.92 ± 0.73	4.41 ± 0.69	4.73 ± 0.66	4.26 ± 0.68	4.75 ± 0.80	4.21 ± 0.70	4.99 ± 0.72	4.45 ± 0.63
LVSD, cm	3.57 ± 0.90	3.02 ± 0.80	3.78 ± 0.81	3.03 ± 0.73	3.72 ± 0.92	3.01 ± 0.79	3.51 ± 0.83	3.02 ± 0.65
LVMi, g/m^2^	119 ± 32.0	107 ± 32.5	120 ± 24.9	100 ± 30.9	111 ± 28.9	102 ± 28.2	95.9 ± 28.6	79.5 ± 24.2
LVEF, %	54 ± 15	58 ± 14	49 ± 14	59 ± 14	47 ± 16	59 ± 11	55 ± 12	59 ± 10
Septal E:e′ ratio	17.4 ± 8.36	19.3 ± 9.19	27.8 ± 15.9	23.9 ± 13.8	19.8 ± 10.4	22.0 ± 11.9	10.5 ± 5.34	11.5 ± 5.99
Transmitral E:A ratio	0.99 ± 0.66	1.09 ± 0.80	0.93 ± 0.54	1.02 ± 0.56	1.24 ± 1.04	1.11 ± 0.71	1.18 ± 0.69	1.16 ± 0.61
SVi, mL/m^2^	38.0 ± 13.5	40.3 ± 14.0	31.6 ± 13.1	37.5 ± 10.8	37.8 ± 12.1	42.8 ± 11.4	40.1 ± 1.9	39.5 ± 10.8

Legend: This table compares the baseline characteristics of all 9189 individuals according to the blinded AI-AAS detection and classification of AS on a sex-specific basis. Whilst more men than women were initially categorised as having guideline-defined severe AS, the original disparity between sexes was reduced when more women than men were recategorized as having severe AS by the AI-AAS. Number of individuals for each variable: Heart rate = 4401 men/3680 women; systolic blood pressure (BP) = 5121 men/2454 women; mean aortic valve (AV) gradient = 3369 men/2994 women; AV area = 2928 men/2648 women; estimated right ventricular systolic pressure (eRVSP)/tricuspid regurgitation (TR) peak velocity = 3379 men/3153 women; left atrial volume index (LAVi) = 3904 men/3306 women; left ventricular diastolic dimension (LVDD) = 3442 men/2642 women; left ventricular systolic dimension (LVSD) = 3393 men/2606 women; left ventricular mass index (LVMi) = 2372 men/2008 women; E:e′ ratio = 4212 men/3445 women; E:A ratio 4253 men/3586 women and stroke volume index (SVi) = 4275 men/3364 women; left ventricular ejection fraction (LVEF).

## 3 Results

### 3.1 Study cohort

Of the main study cohort of 9189 individuals, 4057 (44.2 %) were women (mean age 61.6 ± 18.1 years) and 5132 were men (60.8 ± 17.5 years); p = 0.034. As shown in Fig. 1, 218 (2.4 %) severe AS cases were initially diagnosed by the reporting cardiologist, compared to 376 (4.1 %) cases identified with guideline-defined severe AS by the AI-AAS (a 72 % increase). A further 66 (0.7 %) cases were identified with (an AI-defined) severe AS phenotype that did not satisfy the current guidelines for severe AS. An additional 108 (1.2 %) moderate AS cases were identified. Overall, 550 (6.0 %) cases (1: 1 ratio of men: women) were identified by the AI-AAS as having moderate-to-severe AS. The remaining 8639 cases, comprised 3784 women (41.2 %, 60.4 ± 18.0 years) and 4855 men (60.0 ± 17.4 years) were deemed to have less than moderate AS.

### 3.2 Initial identification/classification of aortic stenosis

Overall, more men (47.3 %) than women (31.9 %) were initially diagnosed with severe AS. These cases had a significantly higher Vmax and AV mean pressure gradient (all p < 0.001). Although there was a statistically significant difference in AVA (p < 0.001), 50 % of the non-severe initial diagnosis met current guidelines based on their AVA. Left Ventricular Mass index (LVMi) was consistently different between men and women, which was significantly higher (p = 0.046 for men and p < 0.001 for women) compared to the AI-AAS identified cases. LVEF was more preserved in men (p = 0.046); whilst in women only, the LVDD was significantly larger in those reported to have severe AS.

Overall, 172/376 (45.7 %) cases with guideline-defined severe AS were not initially classified as having severe AS. This mis-diagnosis/non-detection was more pronounced among women (92/169, 54.4 %) than men (80/207, 38.6 %); adjusted OR 0.21 (95%CI 0.10–0.45; p < 0.001). A correct diagnosis of guideline-defined severe AS also correlated with a higher Vmax (OR 1.03, 95%CI 1.01–1.04 per unit) and smaller AVA (OR 0.02, 95%CI 0.003–0.09 per unit; both p < 0.001), but not according to age, mean AV gradient, LVEF or body mass index (Table 1).

### 3.3 AI-derived identification/classification of aortic stenosis

Table 2 compares the baseline characteristics of 9189 individuals according to the blinded AI-AAS detection and classification of AS on a sex-specific basis. Whilst more men than women were initially categorised as having guideline-defined severe AS (55.1 % versus 44.9 %), the original disparity between sexes was reduced when more women than men were re-categorised as having severe AS by the AI-AAS (1.2 % versus 0.4 %). As expected, those reclassified as guideline-defined severe AS (376 cases) had smaller AVAs (0.89±0.31 cm^2^ in men and 0.85±0.22 cm^2^ in women). This was the most common reason for triggering a severe AS guideline reclassification (209/340 [61.5 %] with AVA recorded). Similarly, both Vmax (3.82±0.79 m/s in men and 3.69±0.9 m/s in women) and mean AV gradient (39.4±15.6 mmHg and 37.0±18.4 mmHg) were above guideline-defined severe AS thresholds in 169/550 (30.7 %) and 160/509 (31.4 %) cases, respectively.

More women than men were likely to be re-categorised as having an AI-defined severe AS phenotype that did not satisfy the guideline criteria for severe AS. Overall, 216 (48.9 %) of 442 women were defined by the AI-AAS as having severe AS. Finally, four cases identified by the AI-AAS as having severe AS had hypertrophic cardiomyopathy (elevated aortic velocities due to left ventricular outflow tract obstruction rather than valvular AS).

### 3.4 Initial clinical management

Table 3 shows the distribution of planned management according to sex in the same 550 cases identified with moderate-to-severe AS described in Table 2. Overall, 70 (25.3 %), 45 (16.2 %) and 2 (0.7 %) men compared to 22 (8.1 %), 46 (16.8 %) and 9 (3.3 %) women were considered for SAVR, TAVR and BAV, respectively. Definitive medical management plans were also documented in 54 (19.5 %) men and 42 (15.4 %) women, in whom guideline-defined severe AS was present in 63.0 % of men and 52.4 % of women. When compared with the valve intervention group, the medical management group were significantly older, had a higher prevalence of atrial fibrillation and left atrial dilatation, higher left ventricular filling pressure and pulmonary systolic pressure, lower ejection fraction (in men only), a higher prevalence of comorbid conditions, including coronary artery disease, hypertension, diabetes, and chronic kidney disease, and a greater use of diuretics and beta-blockers. Whilst dyspnea was the dominant symptom of AS in all cases, it was most prevalent in the medical treatment group (59.3 % of men and 76.2 % of women), with diuretics used in 51.9 % of men and 59.5 % of women within this group.

**Table 3:** Group characteristics according to planned clinical management of AS (N = 550).

	Valve intervention		Medical management		Non-definitive	
	Men (n = 117)	Women (n = 77)	Men (n = 54)	Women (n = 42)	Men (n = 106)	Women (n = 154)
Demographic profile						
Age, years	73.4 ± 9.8	78.3 ± 9.9	76.9 ± 12.0	80.7 ± 8.9	77.0 ± 12.3	76.8 ± 9.9

Clinical profile						
BMI, kg/m^2^	29.0 ± 4.9	28.8 ± 6.4	27.2 ± 5.3	29.9 ± 8.0	27.2 ± 5.3	28.7 ± 7.0
Systolic BP, mmHg	130 ± 22	141 ± 25	129 ± 20	132 ± 21	133 ± 27	140 ± 30
Heart rate, bpm	71 ± 20	72 ± 13	77 ± 17	74 ± 12	70 ± 13	72 ± 13

Aortic valve function/dimension						
Vmax, m/s	4.10 ± 0.61	4.11 ± 0.67	3.17 ± 0.84	3.27 ± 0.81	3.20 ± 0.86	2.99 ± 0.81
Mean AV gradient, mmHg	44.8 ± 13.7	44.5 ± 16.9	28.7 ± 16.0	27.8 ± 16.3	28.5 ± 14.6	24.6 ± 13.8
Valve area, cm^2^	0.85 ± 0.27	0.82 ± 0.26	1.02 ± 0.40	0.95 ± 0.27	1.02 ± 0.30	0.99 ± 0.26

Aortic stenosis category						
Severe AS (guidelines)	108 (92.3 %)	63 (81.8 %)	34 (63.0 %)	22 (52.4 %)	65 (61.3 %)	84 (54.5 %)
Severe AS phenotype (AI-DSA)	2 (1.7 %)	10 (13.0 %)	5 (9.3 %)	9 (21.4 %)	12 (11.3 %)	11 (26.2 %)
Moderate AS phenotype (AI-DSA)	7 (6.0 %)	4 (5.2 %)	15 (27.8 %)	11 (26.2 %)	29 (27.4 %)	42 (27.3 %)

Right ventricular function						
eRVSP, mmHg	35.5 ± 10.3	36.8 ± 11.3	41.3 ± 12.8	41.5 ± 13.4	37.4 ± 12.8	39.9 ± 14.0
TR peak velocity, m/s	2.762 ± 0.43	2.76 ± 0.52	2.96 ± 0.54	2.98 ± 0.52	2.77 ± 0.56	2.90 ± 0.55

Left ventricular function/dimensions						
LAVi, mL/m^2^	45.0 ± 16.6	48.6 ± 22.0	54.0 ± 25.2	55.0 ± 20.4	49.1 ± 21.3	50.0 ± 20.9
LVDD, cm	4.86 ± 0.72	4.42 ± 0.62	4.99 ± 0.82	4.41 ± 0.68	4.84 ± 0.72	4.26 ± 0.73
LVSD, cm	3.45 ± 0.84	3.05 ± 0.80	3.78 ± 1.01	3.07 ± 0.96	3.70 ± 0.88	2.99 ± 0.71
LVMi, g/m^2^	115 ± 30.3	107 ± 29.9	125 ± 34.9	113 ± 42.1	116 ± 28.9	101 ± 28.3
LVEF, %	54 ± 15	58 ± 15	47 ± 18	55 ± 19	53 ± 13	59 ± 11
Septal E:e′ ratio	17.1 ± 7.56	20.5 ± 9.97	22.0 ± 18.2	22.5 ± 13.4	18.2 ± 8.84	20.2 ± 10.3
Transmitral E:A ratio	0.95 ± 0.61	1.03 ± 0.65	1.28 ± 1.03	1.17 ± 0.82	1.04 ± 0.76	1.09 ± 0.77
SVi, mL/m^2^	39.8 ± 13.3	42.3 ± 13.4	33.7 ± 13.1	36.2 ± 14.7	37.2 ± 13.1	40.5 ± 12.6

Risk profile						
Hypertension	52 (44.4 %)	41 (53.2 %)	35 (64.8 %)	29 (69.0 %)	57 (53.8 %)	87 (56.5 %)
Diabetes	27 (23.1 %)	21 (27.3 %)	17 (31.5 %)	14 (33.3 %)	35 (33.0 %)	41 (26.6 %)

Symptoms						
Dyspnoea	58 (49.6 %)	46 (59.7 %)	32 (59.3 %)	32 (76.2 %)	34 (32.1 %)	56 (36.4 %)
Chest pain	27 (23.1 %)	12 (15.6 %)	14 (25.9 %)	8 (19.0 %)	18 (17.0 %)	15 (9.7 %)
Syncope	8 (6.8 %)	5 (6.5 %)	2 (3.7 %)	1 (2.4 %)	5 (4.7 %)	5 (3.2 %)

Clinical profile						
Coronary artery disease	37 (31.6 %)	16 (20.8 %)	26 (48.1 %)	11 (26.2 %)	48 (45.3 %)	24 (15.6 %)
Cerebrovascular disease	2 (1.7 %)	10 (13.0 %)	12 (22.2 %)	8 (19.0 %)	19 (17.9 %)	23 (14.9 %)
Peripheral artery disease	9 (7.7 %)	5 (6.5 %)	7 (13.0 %)	5 (11.9 %)	13 (12.3 %)	7 (4.5 %)
Atrial fibrillation	18 (15.4 %)	14 (18.2 %)	24 (44.4 %)	15 (35.7 %)	28 (26.4 %)	46 (29.9 %)
Chronic pulmonary disease	9 (7.7 %)	5 (53.2 %)	8 (14.8 %)	6 (14.3 %)	13 (12.3 %)	21 (13.6 %)
Cancer/malignancy	5 (4.3 %)	7 (9.1 %)	7 (13.0 %)	6 (13.3 %)	16 (15.1 %)	14 (9.1 %)

Pharmacotherapy						
Diuretic (loop/thiazide)	29 (24.8)	28 (36.4 %)	28 (51.9 %)	25 (59.5 %)	37 (34.9 %)	57 (37.0 %)
ACEi/ARB/ARNi	41 (35.0 %)	35 (45.5 %)	22 (40.7 %)	20 (47.6 %)	43 (40.6 %)	61 (39.6 %)
Beta-blocker	29 (24.8 %)	24 (31.2 %)	27 (50.0 %)	21 (50.0 %)	35 (33.0 %)	56 (36.4 %)
Calcium-antagonist	15 (12.8 %)	11 (14.3 %)	7 (13.0 %)	11 (26.2 %)	29 (27.4 %)	34 (22.1 %)
Mineralocorticoid antagonist	6 (5.1 %)	5 (6.5 %)	5 (9.3 %)	8 (19.0 %)	10 (9.4 %)	18 (11.7 %)
Cholesterol lowering agent	49 (41.9 %)	35 (45.5)	29 (53.3 %)	18 (42.9 %)	55 (51.9 %)	70 (45.5 %)

Legend: This table compares the baseline (see Table 1 for available data) and symptom/management profile (data captured for all cases) according to whether there was a documented plan for aortic valve intervention or medical management or no definitive documentation. Aortic stenosis, AS; body mass index, BMI; blood pressure, BP; aortic valve, AV; estimated right ventricular systolic pressure, eRVSP; tricuspid regurgitation, TR; left atrial volume index, LAVi; left ventricular diastolic dimension, LVDD; left ventricular systolic dimension, LVSD; left ventricular mass index, LVMi; left ventricular ejection fraction, LVEF; stroke volume index, SVi; angiotensin-converting enzyme inhibitors, ACEi; angiotensin II receptor blocker, ARB and angiotensin receptor-neprilysin inhibitor, ARNi.

On an adjusted basis, the probability of valve intervention (138 versus 202 cases in the model) decreased in women (OR 0.54, 95%CI 0.31–0.95), with increasing AVA (OR 0.19, 95%CI 0.06–0.57 per unit) and in the presence of a stroke history (OR 0.32, 95%CI 0.13–0.77), atrial fibrillation/flutter (OR 0.49, 95%CI 0.25–0.98) and hypertension (OR 0.54, 95%CI 0.31–0.95) (all p < 0.05). Conversely, valve intervention was more likely with increasing Vmax (OR 1.02, 95%CI 1.01–1.03; p < 0.001).

Independent of sex, the decision to apply medical management (48 versus 292 cases) was more likely with increasing age (OR 1.04, 95%CI 1.00–1.08 per year) and in the presence of dyspnoea (OR 3.28, 95%CI 1.62–6.61) (both p < 0.05).

Overall, 106 (38.3 %) men and 154 (56.4 %) women had no evidence of definitive management/follow-up. Guideline-defined severe AS was identified in 61.3 % and 54.5 % of these cases. The burden of comorbidities and symptoms were significantly more than those who underwent valve intervention, but less than or comparable to those with intended medical management. This non-definitive status (154 versus 186 “managed” cases in the model) correlated with being a woman (OR 2.09, 95%CI 1.24–3.53), Vmax (OR 0.99, 95%CI 0.98–0.99 per unit), LVEF (OR 1.02, 95%CI 1.00–1.04 per unit), hypertension (OR 2.29, 95%CI 1.32–3.98) and dyspnoea (OR 0.31, 95%CI 0.18–0.54) (all p < 0.05).

Among the 149/376 (39.6 %) cases with guideline-defined severe AS and non-definitive management (Supplementary Table S1), 21/65 (32.3 %) men and 42/84 (50.0 %) women had a major illness/potential contraindication to valve intervention, respectively (adjusted OR for women versus men 2.28, 95%CI 1.18–4.40; p = 0.014). Overall, this left 86/149 (58 %) cases comprising 44 men (77.6 ± 13.9 years) and 42 women (78.1 ± 10.5 years) who had guideline-defined severe AS in whom a major contraindication to valve intervention was not evident. On an adjusted basis, a higher Vmax (OR 0.99, 95%CI 0.98–1.00; p = 0.007) was associated with a reduced likelihood of this being the case, whilst a higher LVEF (OR 1.03, 95%CI 1.01–1.05; p = 0.019) decreased the likelihood, with no sex-based differences observed in this regard.

### 3.5 Potential AI-AAS redirection to surgical management

**Fig. 2:**
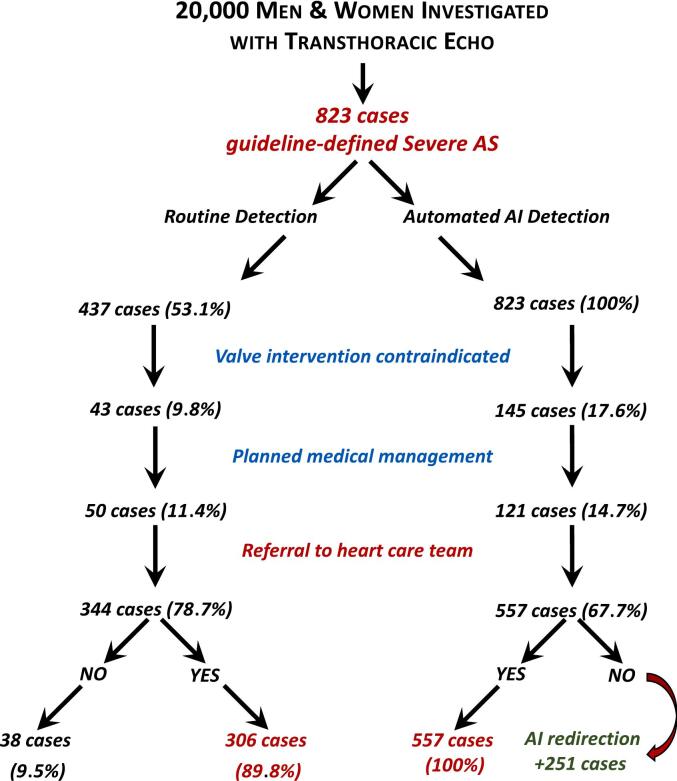
Actual versus potential management of guideline-severe AS. Legend: Artificial intelligence, AI; aortic stenosis, AS; aortic valve replacement, AVR. 1. Strom JB, Playford D, Stewart S, Strange G. An Artificial Intelligence Algorithm for Detection of Severe Aortic Stenosis: A Clinical Cohort Study. JACC Adv 2024;3:101176.

Based on the real-world pattern of recognition and then management of guideline-defined severe AS cases described above, per 10,000 women and men undergoing transthoracic echo, overall, a routinely applied AI-AAS had the potential to redirect 104/420 (24.8 %) and 38/403 (9.4 %) cases, respectively, of guideline-defined AS to more definitive treatment (Fig. 2).

## 4 Discussion

To our knowledge, this is the first study to comprehensively assess the clinical characteristics and consequences of individuals presenting with severe AS according to their initial “standard of care” diagnosis and intended management versus that derived from an AI interpretation of the same echocardiographic data. This two-layered approach highlighted important issues in the real-world management of severe AS. These include the potential “unconscious clinical bias” in recognising severe AS patients (especially women) who might most benefit from life-saving treatment. When subsequently applied to the same echocardiography data, the AI-AAS immediately corrected a strong male bias that flowed into the intended clinical management of severe AS cases and indeed later management. Specifically, on an adjusted basis, women were still two-fold less likely to be considered for definitive valve intervention, whilst two-fold more likely to have ill-defined management. A higher prevalence of contraindications to valve intervention among women did not fully explain the sex-specific disparities in management we uncovered. Critically, reflecting a chain of clinical-decision making, those initially diagnosed with severe AS were more than three times more likely to undergo definitive AV intervention. Beyond observed sex-based differentials, it appears that the principal driver for the routine diagnosis of severe AS was the transaortic gradient rather than the AVA (hemodynamic bias), possibly due to clinical suspicion that an AVA <1.0 cm^2^ is due to LVOT measurement errors rather than severe AS. The AI-AAS ignores all LVOT data in its determination, thus minimising the AVA measurement errors and enhancing diagnosis of severe AS.

The importance of recognising those at risk of dying from AS cannot be overstated, and applying more timely, life-saving interventions is also pivotal to improving health outcomes [2]. Studies from a range of health systems have consistently demonstrated that under-treatment of severe AS is associated with high mortality [21]. Low-gradient AS, which can be technically challenging to diagnose, is particularly subject to variation in clinical interpretation of severity and under-represented in those selected for AV intervention [22] In this context, our novel approach meets the growing calls for automated echocardiographic reporting to reliably and consistently identify individuals with AS who would benefit from specialist valve team management [23,24]. Previously, a range of AI techniques have been applied to expedite the timely diagnosis and management of severe AS. This includes cardiac auscultation augmented by a digital AI-assisted stethoscope that has potential to identify those requiring cardiac imaging [11]. More sophisticated technology involving non-invasive inertial sensors [12] has also shown promising outcomes [24,25,31]. We have progressively refined our approach to identifying AS from routinely reported echocardiograms by applying newer machine-learning techniques [17,18]. They have also helped us understand the progressive nature of AV disease (involving valve calcification and myocardial fibrosis), as well as understanding the normalisation of AV function following valve intervention by applying topological mapping techniques to echocardiographic measurements [26]. There is increasing potential, therefore, to apply machine-learning techniques to streamline the identification of high-risk AS cases in routine clinical care [27]. However, given these new techniques are mainly “demonstrated” using retrospective data, prospective clinical trials are urgently needed to confirm clinical and cost-benefits [28].

Although trained using data from a large multicultural Australian cohort [16], the AI-AAS has been tested in a North American population with greater exposure to specific ethnic groups, showing consistent outcomes [29]. The AI-AAS also identified patients with hypertrophic obstructive cardiomyopathy with high LVOT gradients. Although these few patients did not have a primary diagnosis of AS, highlighting these at-risk individuals in need of clinical review is consistent with the system's goal of decision-support and not to fully automate the diagnosis of severe AS. As in our study cohort, some individuals with severe AS have other illnesses, (e.g., dementia, advanced general frailty, or another cardiac condition) that limit interventional options. Further, illnesses such as thyrotoxicosis and anaemia could have an impact on the valve gradient evaluated by the AI-AAS. Although the AI-AAS reliably identifies low-gradient severe AS, this form of AS is typically associated with other cardiac diseases, with prognosis affected by both the AS and the underlying cardiac pathology. We would emphasise, therefore, that the AI-AAS is not intended to replace clinical decision-making. Instead, it is designed as a standalone plug-in, or directly incorporated into echocardiographic reporting systems, thereby triggering an automated alert to the presence severe AS and identifying individuals, particularly women, who require further consideration for valve intervention. Critically, no modification of standard echocardiographic imaging or acquisition workflow is required. An automated Application Programming Interface (API) has been created by the developing company (ECHO IQ Ltd; Echo IQ Connect) which allows simple integration with vendor agnostic implementation that is quick, simple and easy to install in all digital labs with a PACS system in place.

In conclusion, when compared to its routine detection, we have demonstrated that a novel AI-AAS can identify more individuals with guideline-defined severe AS who would benefit from more definitive management. Consistent with calls from major cardiac societies [30], an objective AI-driven alert can be automatically generated as an integral aspect of echocardiography reporting, without increasing clinical workloads. With an evolving evidence base around who might benefit from more proactive treatment, pending more definitive clinical trials, this and other machine-learning tools have the potential to ensure more people with severe forms of AS receive life-saving care.

The following is the supplementary data related to this article.

Supplementary Table S1 Sex-specific characteristics of those with guideline-defined, severe-AS non-definitive management (N = 149). Supplementary Table S1

## Ethical statement

This study conforms to the “Standards for Reporting Diagnostic accuracy studies” (STARD) and RECORD guidelines for the reporting of routine clinical data. Ethics approval was obtained under the auspices of the National Echo Database of Australia (NEDA – ACTRN12617001387314).

## CRediT authorship contribution statement

**Geoffrey A. Strange:** Writing – review & editing, Supervision, Investigation, Funding acquisition, Conceptualization. **Michael P. Feneley:** Writing – review & editing, Supervision, Project administration, Investigation. **David Prior:** Writing – review & editing, Supervision, Project administration, Investigation. **David Muller:** Writing – review & editing, Supervision, Investigation. **Prasanna Venkataraman:** Writing – review & editing, Project administration, Investigation, Data curation. **Yiling Situ:** Writing – review & editing, Project administration, Investigation, Data curation. **Simon Stewart:** Writing – review & editing, Writing – original draft, Validation, Software, Methodology, Investigation, Formal analysis. **David Playford:** Writing – review & editing, Supervision, Methodology, Investigation, Funding acquisition, Conceptualization.

## Declaration of competing interest

GS and DPL are the Co-Principal Investigators and Directors of NEDA (a not-for-profit research entity). NEDA has received investigator-initiated funding support from Novartis Pharmaceuticals, Pfizer Pharmaceuticals, ECHO IQ, Bristol-Meyer Squibb, Edward Lifesciences, and the Western Australian Government in the past three years. As Research Director, SS has received consultancy fees from NEDA. SS, DPL, and GS have previously received consultancy/speaking fees from Edwards Lifesciences and Medtronic (DPL and GS). GS and DPL receive consultancy fees from ECHO IQ Pty Ltd.

## Data Availability

Requests for anonymised aggregated cohort data can be submitted to the corresponding author for institution consideration.
