# Supplementary material for: Detection of severe aortic stenosis by clinicians versus artificial intelligence: A retrospective clinical cohort study
Source: Am Heart J Plus. 2024 Nov 22;48:100485. doi: 10.1016/j.ahjo.2024.100485 (PMC11648779; doi:10.1016/j.ahjo.2024.100485)
Supplement: Supplementary Table S1 — Sex-specific characteristics of those with guideline-defined, severe-AS non-definitive management (N = 149). [file mmc1.docx]

**Supplementary Material – Table of Content**

1. Supplementary Table S1: Sex-specific characteristics of those with guideline-defined, severe-AS non-definitive management (N=149)

**Supplementary Table S1: Sex-specific characteristics of those with guideline-defined, severe-AS non-definitive management (N=149)**

|  | **Men**  **N=65** | **Women**  **N=84** |
| --- | --- | --- |
| ***Demographic Profile*** | | |
| Age, years | **78.0±12.1** | **77.2±12.2** |
| ***Risk Profile*** | | |
| BMI, kg/m^2^ | **27.8±4.81** | **28.6±6.23** |
| Hypertension, % | **37 (56.9%)** | **59 (70.2%)** |
| Diabetes, % | **24 (36.9%)** | **27 (32.1%)** |
| ***Symptoms*** | | |
| Angina/Chest Pain, % | **12 (18.5%)** | **7 (8.3%)** |
| Dyspnoea, % | **3 (4.6%)** | **4 (4.8%)** |
| Syncope, % | **20 (30.8%)** | **38 (45.2%)** |
| ***Clinical Profile*** | | |
| Vmax, m/s | **3.48±0.84** | **3.27±0.86** |
| LVEF, % | **54.5±12.3** | **59.5±12.3** |
| Coronary artery disease, % | **30 (46.2%)** | **14 (16.7%)** |
| Atrial fibrillation, % | **20 (30.8%)** | **27 (32.1%)** |
| Hypertrophic cardiomyopathy, % | **2 (3.1%)** | **2 (2.4%)** |
| Cardiac amyloidosis, % | **3 (4.6%)** | **3 (3.6%)** |
| Congenital heart disease, % | **2 (3.1%)** | **2 (2.4%)** |
| Stroke, % | **15 (23.1%)** | **15 (17.9%)** |
| Peripheral artery disease, % | **8 (12.3%)** | **3 (3.6%)** |
| Chronic obstructive pulmonary disease, % | **6 (9.2%)** | **17 (20.2%)** |
| Cancer, % | **11 (16.9%)** | **6 (7.1%)** |
| Other serious illness, % | **21 (32.3%)** | **40 (47.6%)** |
